# Disseminated orbital actinomycetoma: A case report

**DOI:** 10.4103/0301-4738.58474

**Published:** 2010

**Authors:** Nita Umesh Shanbhag, Sumita Karandikar, Pooja Anil Deshmukkh

**Affiliations:** D. Y. Patil Medical College and Research Center, Nerul, Navi Mumbai, India

**Keywords:** Actinomycetoma, eyelid, nocardia, orbit, scalp sinuses

## Abstract

Mycetoma is a chronic granulomatous infection. Lower extremities are commonly involved. A 20-year-old male came with complaints of multiple sinuses on scalp, left eyelid swelling with a sinus and dystopia, since one year. On examination there was relative proptosis in left eye of 2 mm. Computed tomography scan showed soft tissue swelling of the pre-septal area of the left upper eyelid with orbital involvement. Magnetic resonance imaging showed increased left orbital volume and evident dystopia. Microbiology testing of the erosive scalp and lid lesions showed genus Nocardia, suggestive of actinomycetoma. This case is presented as it shows an unusual involvement of the orbit.

Mycetoma is a chronic granulomatous infection affecting the skin, subcutaneous tissues, and bone. The causative agents include bacteria (actinomycetoma), fungi (eumycetoma), which gain entry to the skin by traumatic inoculation.[1] Foot is the commonest site followed by the upper extremity.[2] Involvement of the perineum being third in the order of frequency and the fourth commonest site is the scalp.[3]

We are reporting this case because orbital involvement of actinomycetoma is very unusual. In our review of the literature, we did not come across any such case.

## Case Report

**Figure 1:**
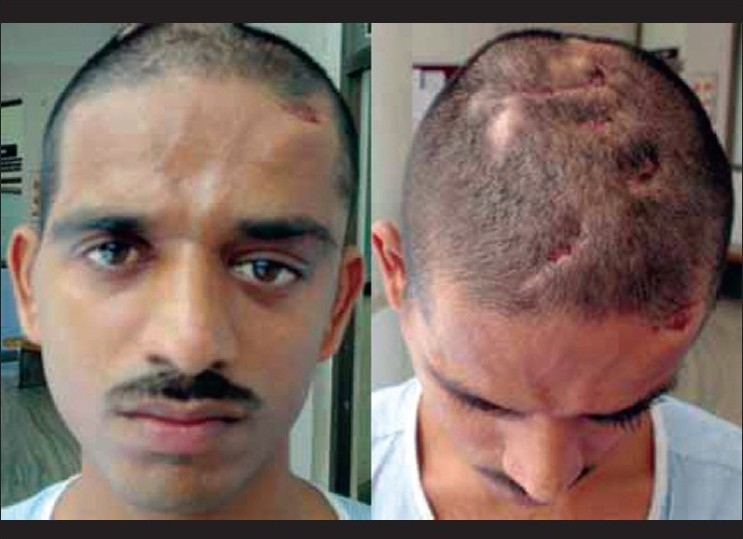
Clinical picture showing multiple draining sinuses of scalp and left upper lid with dystopia

A 20-year-old male came with complaints of multiple sinuses over the region of the scalp for a period of one year. He gave a similar history of sinus over the left eyelid since a year, with dystopia for the last six months. The dystopia was gradually progressive, painless, with no diminution of vision or diplopia [Fig. 1]. He further gave history of lacerated wound being sutured over the left eyebrow five years back, following fall on the left forehead.

**Figure 2:**
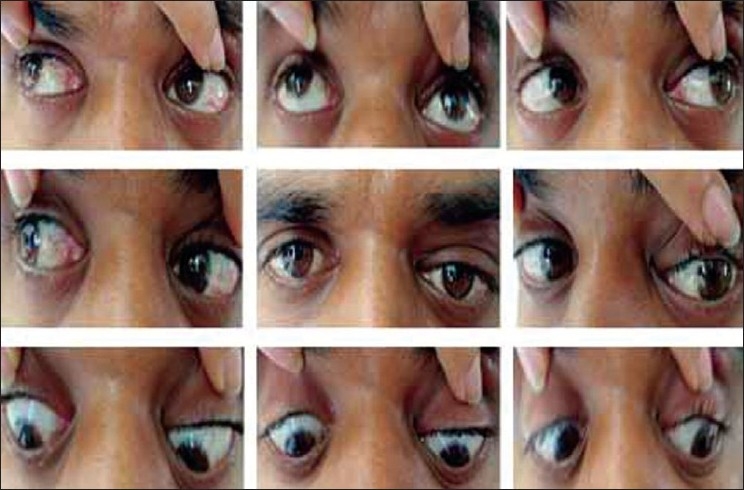
Extraocular movements showing mild restriction in the left gaze

On examination of the eyes, vision was 20/20 in both eyes. All findings in right eye were within normal limits. The left eye showed dystopia of 20 prism diopters (Δ) downwards and 10 Δ outwards on prism bar test. On exophthalmometry there was relative proptosis in left eye of 2 mm. The extraocular movements were within normal limits except for restriction in left gaze [Fig. 2]. The left eyelid showed a diffuse swelling with a discharging sinus. The anterior segment, pupil, fundus and diplopia charting were within normal limits. General examination showed multiple sinuses on the scalp which were draining white grains.

**Figure 3:**
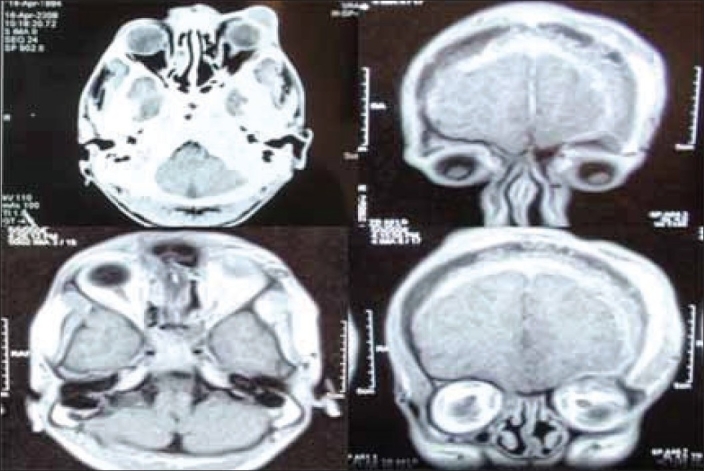
Computerized Tomography Scan showing involvement of the Left eyelid, orbit with extradural extension

**Figure 4:**
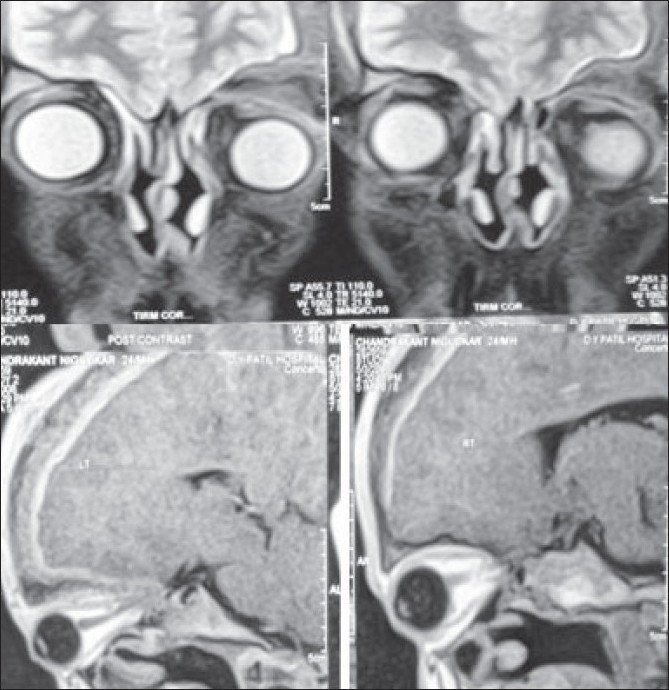
Magnetic resonance imaging showing dystopia and increase in left orbit volume

Apart from leucocytosis all routine investigations were within normal limits. Computer tomography (CT) scan of the brain revealed extradural invasion. CT of the orbit showed soft tissue swelling of the pre-septal area of the left upper eyelid with orbital involvement [Fig. 3]. Magnetic resonance imaging (MRI) showed increased left orbital volume and evident dystopia [Fig. 4].

**Figure 5:**
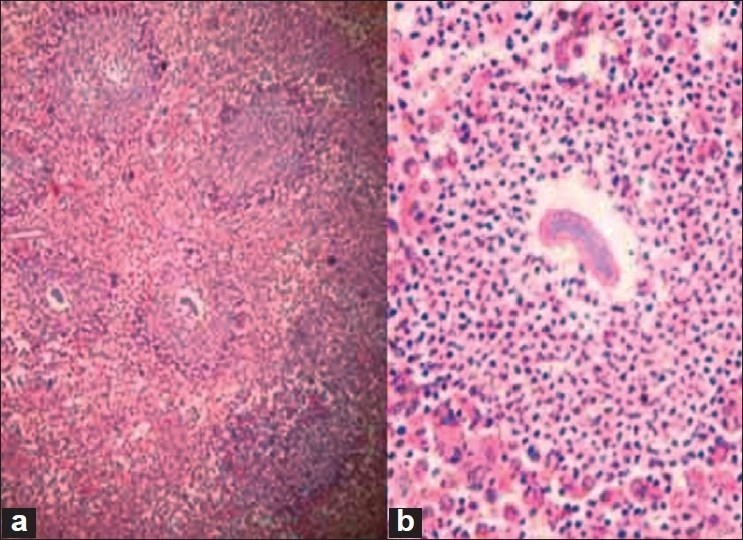
Histopathology showing eosinophilic granule exhibiting a Splendore-Hoeppli Phenomenon Stain: Hematoxylin and Eosin stain Magnification: (a) 10×, (b) 40×

Microbiology testing of the erosive scalp and lid lesions showed pus cells and *Nocardia Asteroides / Nocardia Brazilensis bacillus*, highly suggestive of actinomycetoma. Histopathology revealed lympho-histiocytic infiltration with focal abscess formation. Inset shows an eosinophilic granule exhibiting a Splendore-Hoeppli phenomenon with a background of neutrophils [Fig. 5].

We started treatment with intravenous (IV) streptomycin 80 mg twice daily and tab. cotrimoxazole DS once daily for six weeks in intensive phase. Being a disease resistant to treatment, frequent change in antibiotics is desirable to avoid development of drug resistance. The patient was put on maintenance phase of tab. cotrimoxazole DS once daily and tab. rifampicin 600 mg once daily. He responded well to treatment with healing of sinuses and reduction in dystopia

## Discussion

Mycetoma was first recognized as a clinical entity in Madurai (South India) as madhura foot, but now it is known to be prevalent in other countries.[2] The etiological factors involved in causation are multiple, in the form of thorn prick, trauma leading to ulceration, blunt trauma and the wicks.[3]

Actinomycetoma is caused by actinomycetes which include the genera *Nocardia*, *Actinomyces*, and *Streptomyces*. The members of the genus *Nocardia* are filamentous gram-positive bacteria that are ubiquitous soil saprophytes.[4,5]

The genus *Nocardia* comprises several species of clinical importance. Among these, *N. Brasiliensis* is the main pathogenic organism for primary cutaneous infection, followed by *N. Asteroides*, which usually causes fulminant systemic infection.[6] The incidence of Nocardial mycetoma in Indian reports varies from 5.2-35%.[7,8]

They gain entry into the skin through traumatic inoculation causing localized infection that is focally aggressive but does not typically disseminate. Early diagnosis and treatment can dramatically affect morbidity associated with this condition. Thus, it is important for clinicians to be aware of this disorder's clinical presentation, methods available for diagnosis and the treatment options. Clinically, patients experience formation of erythematous papulonodules with drainage of purulent material and sinus tract formation slowly spreading in a sporotrichoid fashion. Ultimately, fibrosis and destruction of underlying soft tissue and bone will ensue.[4,5]

Previously a case of isolated intracranial granuloma by actinomadura pelletieri has been reported by Kumar *et al*. This patient had a scalp injury following which she developed a swelling and a discharging sinus. Below the scalp lesion there was osteomyletis of skull bone and collection of pus and infected granulation tissue between the fascial planes of the scalp.[6] Cervicofacial actinomycosis constitutes 41-55% of actinomycotic lesions in various studies. Today it is a rare disease, secondary to mucosal trauma or dental problems, wherein they invade the soft tissues of the face and neck. The classic lesion is seen in the submandibular or parotid region. The spread is usually by contiguity through the soft tissue rather than the hematogenous or lymphatic route.[7] Scalp mycetoma with *N. Asteroides* as causative organism was reported by Maiti *et al.,* but there was no deeper involvement in this case.[8]

### Spread of the Disease

Mycetoma, especially when caused by *N. brasiliensis*, has a propensity to involve the underlying bone, and osteolytic changes are frequently observed radiologically. Compressive myelopathy has been reported as a sequel to bone involvement underlying a mycetoma caused by *N. brasiliensis*.[9] This suggests that mycetomas usually spread by direct contiguity showing lid lesion to be the primary focus from which there has been orbital invasion through facial planes.

Primary cutaneous nocardiosis has been reported in patients with HIV infection, lymphoma, Cushing's syndrome or organ transplantation. The clinical severity and course of the disease seem to remain unaltered in this group of patients. The spectrum of causative organisms is similar to that in otherwise healthy individuals. This suggests that intracranial involvement can occur in a healthy individual.[10]

Demonstration of the organism from clinical specimens like granules, pus or aspirated fluid from an unruptured nodule by Gram stain and modified Kinyoun stain is the mainstay of diagnosis. Gram-positive and acid-fast, thin, beaded, branching filaments are the characteristic appearance of the organism. Identification of the *Nocardia* species by culture is a tedious process. The organism is slow-growing and it may take up to two to three weeks for isolation from a clinical specimen.[11] A reaction pattern of granulomatous inflammation, abscess formation, and fibrosis is typical, but non-diagnostic. The identification of characteristic “sulfur granules” or “grains,” which contain the infectious organisms, confirms the diagnosis of type of mycetoma. Grains from *eumycetomas* are larger and black or white-colored seen with the naked eye. *Actinomycetoma* grains are white to yellow in color, not seen with naked eye.

The organisms of actinomycetoma show a central matted appearance often surrounded by a peripheral, radial deposition of intensely eosinophilic material termed a Splendore-Hoeppli reaction.[12,13] Imaging techniques of plain films, CT scan, and MRI allow the clinician to determine the degree of involvement of underlying structures. Imaging findings provide valuable information that helps to plan the aggressiveness of treatment. The likelihood of mycetoma extension to bone increases with a longer duration of disease.

Eumycetomas often respond poorly to pharmacologic therapy with antifungal agents. Surgical intervention in the form of debridement of infected tissue with wide margins is necessary to prevent recurrence. Sulfonamides have been the mainstay of treatment for nocardial actinomycetoma for over 50 years. The duration of treatment is dictated by the clinical response to medication, but reported cure rates are 60-90% with a mean duration of therapy greater than one year.[14] A recent study suggested a modified two-step treatment for actinomycetoma. It consists of an intensive phase (Step 1) with gentamicin, 80 mg twice daily, intravenously and cotrimoxazole, 320/1600 mg twice daily orally for four weeks. This was followed by a maintenance phase with cotrimoxazole and doxycycline, 100 mg twice daily till all sinuses healed completely. The treatment is continued for five to six months.[15] Welsh proposed a two-step schedule in the treatment of actinomycotic mycetomas:

It consists of four injections of penicillin and two of gentamicin daily, in the initial intensive phase of five to seven weeks. Maintenance phase with amoxicillin / doxycycline and cotrimoxazole till two to five months after complete healing.[16]

The message to carry home is avoid inoculation of the organism during primary suturing. Wound cleaning with disinfectants and optimal tissue debridement with complete removal of the necrotic tissue is the dictum.
